# Supplementary material for: HIVprotI: an integrated web based platform for prediction and design of HIV proteins inhibitors
Source: J Cheminform. 2018 Mar 9;10:12. doi: 10.1186/s13321-018-0266-y (PMC5845081; doi:10.1186/s13321-018-0266-y)
Supplement: Supplementary file 2 — Additional file 2. Source code of HIVProtI web server. [file 13321_2018_266_MOESM2_ESM.zip › HIVprotI_Source-code/hivprotI/weka-3-6-6/doc/weka/core/matrix/package-tree.html]

weka.core.matrix Class Hierarchy


---


|  |  |  |
| --- | --- | --- |
| **Overview** \| **Package** \| Class \| **Tree** \| **Deprecated** \| **Index** \| **Help** \| **Weka's home** | |  |
| **PREV**   **NEXT** | **FRAMES**    **NO FRAMES**     **All Classes** |  |


---


## Hierarchy For Package weka.core.matrix

**Package Hierarchies:**: All Packages

---

## Class Hierarchy

- java.lang.Object
  - weka.core.matrix.**CholeskyDecomposition** (implements weka.core.RevisionHandler, java.io.Serializable)- weka.core.matrix.**DoubleVector** (implements java.lang.Cloneable, weka.core.RevisionHandler)- weka.core.matrix.**EigenvalueDecomposition** (implements weka.core.RevisionHandler, java.io.Serializable)- java.text.Format (implements java.lang.Cloneable, java.io.Serializable)
          - java.text.NumberFormat
            - java.text.DecimalFormat
              - weka.core.matrix.**ExponentialFormat** (implements weka.core.RevisionHandler)- weka.core.matrix.**FlexibleDecimalFormat** (implements weka.core.RevisionHandler)- weka.core.matrix.**FloatingPointFormat** (implements weka.core.RevisionHandler)
          - weka.core.matrix.**IntVector** (implements java.lang.Cloneable, weka.core.RevisionHandler)- weka.core.matrix.**LinearRegression** (implements weka.core.RevisionHandler)- weka.core.matrix.**LUDecomposition** (implements weka.core.RevisionHandler, java.io.Serializable)- weka.core.matrix.**Maths** (implements weka.core.RevisionHandler)- weka.core.matrix.**Matrix** (implements java.lang.Cloneable, weka.core.RevisionHandler, java.io.Serializable)- weka.core.matrix.**QRDecomposition** (implements weka.core.RevisionHandler, java.io.Serializable)- weka.core.matrix.**SingularValueDecomposition** (implements weka.core.RevisionHandler, java.io.Serializable)

---


|  |  |  |
| --- | --- | --- |
| **Overview** \| **Package** \| Class \| **Tree** \| **Deprecated** \| **Index** \| **Help** \| **Weka's home** | |  |
| **PREV**   **NEXT** | **FRAMES**    **NO FRAMES**     **All Classes** |  |


---
